# Decreasing Prevalence of the Full Metabolic Syndrome but a Persistently High Prevalence of Dyslipidemia among Adult Arabs

**DOI:** 10.1371/journal.pone.0012159

**Published:** 2010-08-13

**Authors:** Nasser M. Al-Daghri, Omar S. Al-Attas, Majed S. Alokail, Khalid M. Alkharfy, Shaun Louie B. Sabico, George P. Chrousos

**Affiliations:** 1 Biochemistry Department, College of Science, King Saud University, Riyadh, Kingdom of Saudi Arabia; 2 Clinical Pharmacy Department, College of Pharmacy, King Saud University, Riyadh, Kingdom of Saudi Arabia; 3 Division of Endocrinology, Metabolism and Diabetes, University of Athens Medical School, Children's Hospital Aghia Sophia, Athens, Greece; Karolinska Institutet, Sweden

## Abstract

A decade has passed since metabolic syndrome (MetS) was documented to be highly prevalent in the kingdom of Saudi Arabia. No follow-up epidemiologic study was done. This study aims to fill this gap. In this cross-sectional, observational study, a total of 2850 randomly selected Saudi adults aged 18–55 years were recruited. Subjects' information was generated from a database of more than 10,000 Saudi citizens from the existing Biomarkers Screening in Riyadh Program (RIYADH Cohort), Saudi Arabia. Anthropometrics included body mass index (BMI), blood pressure, as well as waist and hip circumferences. Fasting blood glucose and lipid profile were determined using routine laboratory procedures. The definition of ATP-III (NHANES III) was used for the diagnosis of the full MetS. The overall prevalence of complete MetS was 35.3% [Confidence-Interval (CI) 33.5–37.01]. Age-adjusted prevalence according to the European standard population is 37.0%. Low HDL-cholesterol was the most prevalent of all MetS risk factors, affecting 88.6% (CI 87.5–89.7) and hypertriglyceridemia the second most prevalent, affecting 34% (CI 32.3–35.7) of the subjects. The prevalence of the full MetS decreased from previous estimates but remains high, while dyslipidemia remains extremely high, affecting almost 90% of middle-aged Arabs. Screening for dyslipidemia among Saudi adults is warranted, especially among those most at risk. Scientific inquiry into the molecular causes of these manifestations should be pursued as a first step in the discovery of etiologic therapies.

## Introduction

Metabolic syndrome (MetS), which is the clinical term used for the co-occurrence of several cardiovascular risk factors, is quite prevalent in many developed nations. Al-Nozha and colleagues previously reported that MetS was almost 40% among adults in a kingdom-wide sample population taken from 1995–2000 [1]. Since then, other epidemiologic studies conducted within the Arab Peninsula confirmed the same high prevalence [2]–[4]. Aside from the conventional risk factors for MetS which include over nutrition and sedentary lifestyle, other risk factors peculiar to the Arab population have been identified. These include acute post cessation smoking [5], and metabolic abnormalities such as altered adipocytokine levels [6], particularly leptin [7], and homocysteine [8]. A decade has passed, however, and no follow-up epidemiologic study was done in the Saudi Arab population to determine whether the epidemic has been addressed properly. This study aims to determine the prevalence of MetS in a cohort of adult Saudis living in urban areas and to identify which are the most prevalent MetS components.

## Materials and Methods

### Subjects

In this cross-sectional community-based study, a total of 2,850 Saudi adults aged 18–55 years were included. Subjects were part of the capital-wide Biomarker Screening in Riyadh (BSR), a collaborative effort between the Biomarkers Research Program (BRP) of King Saud University (KSU) and the Ministry of Health in Riyadh, Kingdom of Saudi Arabia (RIYADH Cohort). Ethical approval was obtained from the College of Medicine Research Center Ethics Committee of KSU, Riyadh, KSA. In brief, BSR was launched to identify and employ novel biomarkers of chronic non-communicable diseases, including diabetes mellitus (DM), cardiovascular diseases (CVD), and obesity, among consenting Saudis. Subjects were recruited randomly from different Primary Health Care Centers (PHCC) across Riyadh. No expatriates were included in the conduct of this study. Each participating subject submitted a signed consent form, a general questionnaire containing demographic, past and present medical history, as well as diet information from the food frequency questionnaire. Subjects' information was taken from the existing database of more than 10,000 subjects (RIYADH Cohort).

### Anthropometric measurements

Consenting adults were invited to their respective PHCC after a 10 h overnight fasted state. Anthropometric measurements included height (cm), weight (cm), waist (cm) and hip (cm) circumferences and were taken and noted by trained nurses. Systolic and diastolic blood pressures were taken twice with a 15-minute interval. The average of both readings was recorded. Body mass index (BMI) was calculated as weight (kg) over height (m^2^).

### Biochemical measurements

Blood was drawn, centrifuged and processed in the same day. Both whole blood and serum were placed in plain polystyrene tubes and delivered to BRP for storage at−2°C. Fasting blood glucose, triglycerides, and HDL cholesterol were quantified using routine laboratory analysis (Konelab, Finland). This biochemical analyzer (Konelab) was calibrated routinely prior to the analysis of all serum samples using quality control samples provided (Thermo Fisher Scientific, Finland). Glucose test method employed glucose oxidase and modified Trinder color reaction catalyzed by the enzyme peroxidase. Triglycerides test method employed hydrolysis, phosphorylation and oxidation leading to the formation of quinoeimine dye which is absorbed at 510 nm. HDL-cholesterol test method employed homogenous enzymatic colorimetric test by cholesterol oxidase coupled with poly(ethylene-glycol) to the amino groups. Measurement ranges were as follows: glucose (0.3–20.0 mmol/L); triglycerides (0.05–11.0 mmol/L), HDL-cholesterol (0.16–2.80 mmol/L). All samples fell within normal detection limits. Standard International Units (mmol/L) were used to record the results. For validation purposes, randomized samples were sent to a reference lab within the kingdom (King Faisal Specialist Hospital and Research Center, Riyadh, KSA).

### Definition of MetS

The definition of MetS used was according to the National Cholesterol Education Program-Third Adult Treatment Panel (NCEP ATP III), three or more of the following criteria must be fulfilled: fasting blood glucose level ≥5.6 mmol/l; blood pressure ≥130/85 mm Hg; triglycerides ≥1.7 mmol/l; HDL cholesterol <1.03 mmol/l for men and <1.29 mmol/l for women; and waist circumference >102 cm for men and >88 cm for women [9].

### Data Analysis

Statistical analysis was done using the Statistical Package for Social Sciences (SPSS) version 11.5 (Chicago, Illinois). Continuous variables are shown as mean ± standard deviation. Age adjustment was done with a European standard population. Frequencies were presented as percentage (confidence-interval). MetS prevalence was presented as overall prevalence, age-adjusted prevalence and prevalence according to age.

## Results

**Figure 1:**
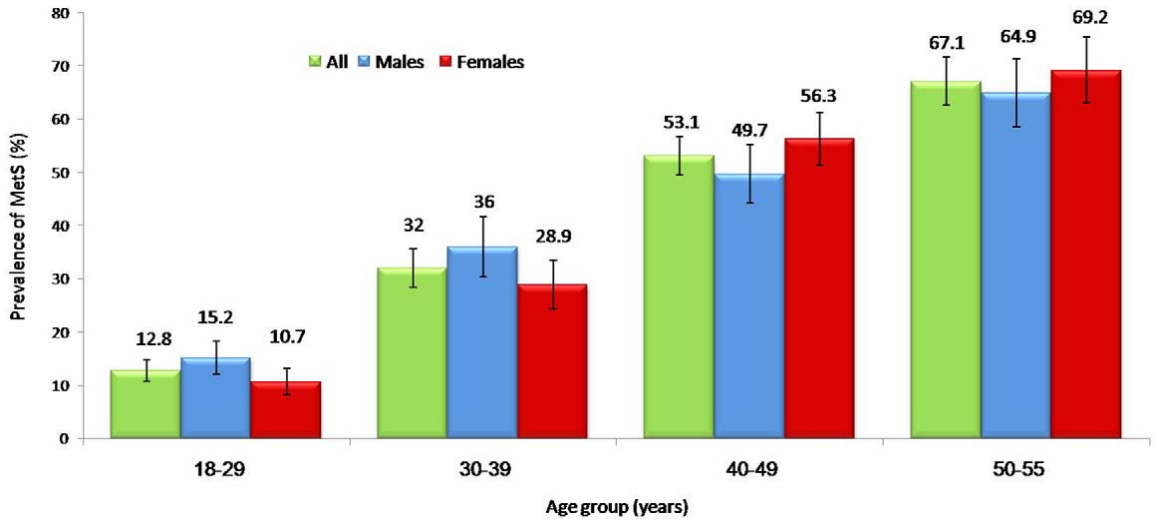
Prevalence of MetS in males and females according to age groups (adult criteria NCEP ATP III [**9**]).

**Figure 2:**
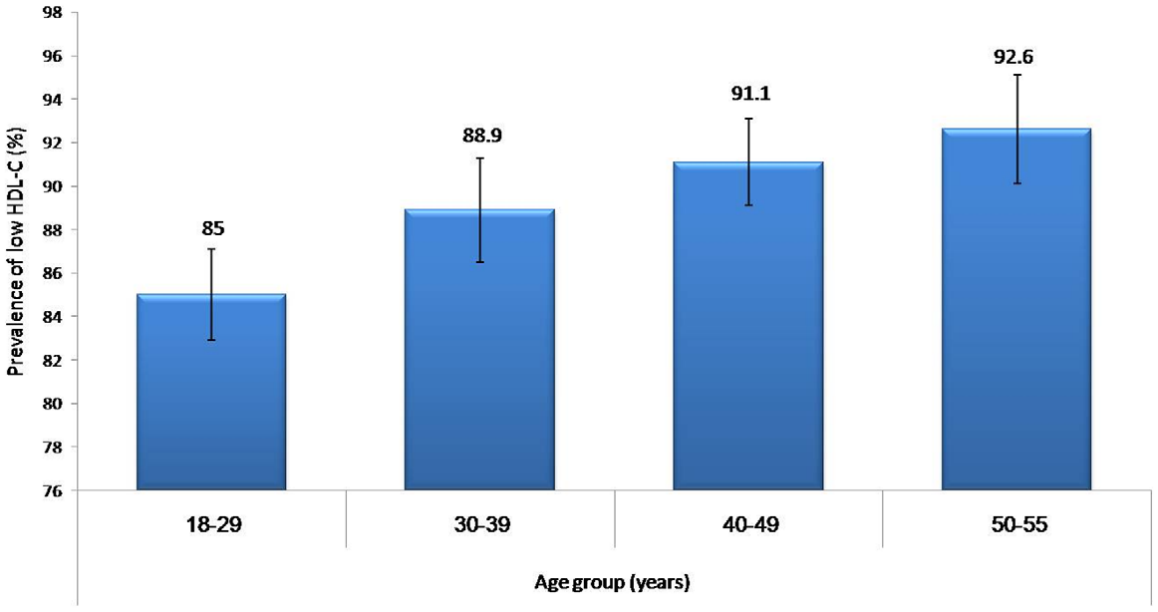
Prevalence of low HDL-cholesterol stratified according to age (adult criteria NCEP ATP III [**9**]).

**Figure 3:**
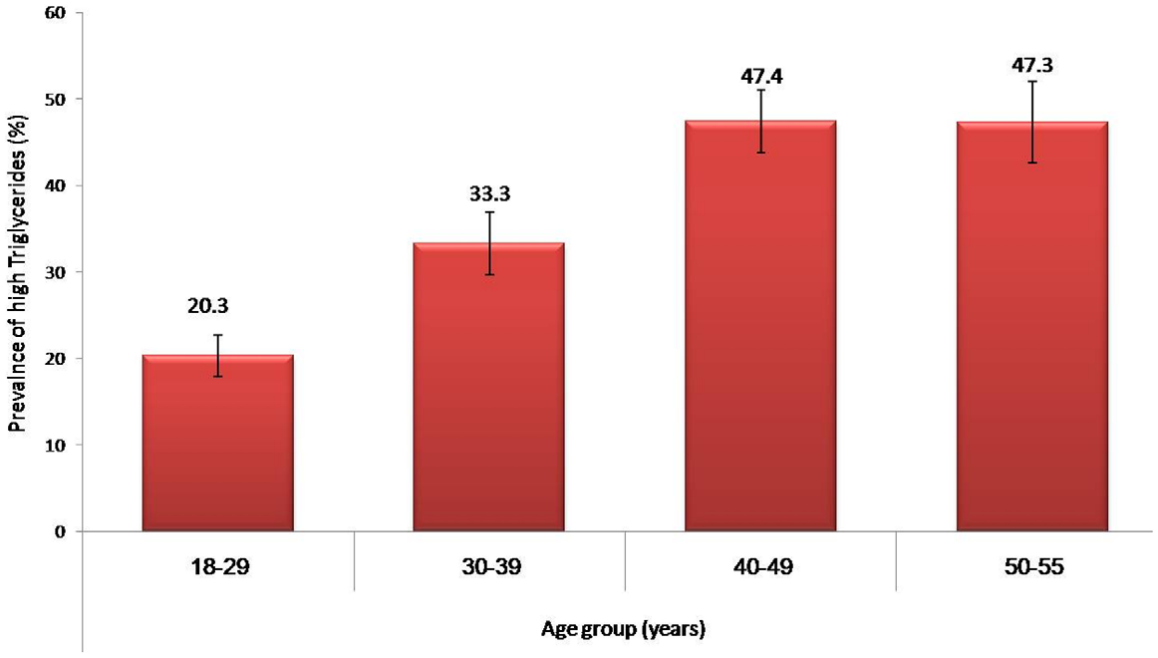
Prevalence of hypertriglyceridemia stratified according to age (adult criteria NCEP ATP III [**9**]).

The over-all prevalence of MetS was 35.3% (CI 33.5–37.01), while an age adjusted prevalence was almost 37.0%. Low HDL-cholesterol remains the most common MetS component with almost 9 out of 10 [88.6% (CI 87.5–89.7)] of subjects affected followed by hypertriglyceridemia with an over-all prevalence of 34% (CI 32.3–35.7) (Table 1). Males had significantly higher prevalence of high blood pressure, impaired fasting glucose and triglycrides (*p*-values 0.004, 0.001<0.001 respectively) while females had significantly higher prevalence of abdominal obesity (*p*-value <0.001). Figure 1 shows the alarmingly increasing prevalence of MetS with age, with the age group 50–55 years harboring MetS in almost 6 out of 10 adults. Figures 2 and 3 show the persistent high prevalence of low-HDL-cholesterol and hypertriglyceridemia even after stratification for age, respectively.

**Table 1:** Prevalence of Individual Components and of the Full MetS in the Urban Saudi Arab Population (Adult criteria NCEP ATP III [9]).

	*Total*	*Males*	*Females*	*P-Value*
*N*	2850	1335	1515	
***Low HDL-Cholesterol***	88.6 (87.5–89.7)	89.4 (87.8–91.0)	87.8 (86.2–89.4)	0.168
***High Blood pressure***	25.4 (23.7–27.1)	28.2 (25.6–30.8)	23.1 (20.9–25.3)	0.004
***Impaired Fasting Glucose***	29.9 (28.3–31.5)	33.0 (30.5–35.5)	27.1 (24.9–29.3)	0.001
***Elevated Triglycerides***	34.0 (32.3–35.7)	42.0 (39.4–44.6)	27.0 (24.8–29.2)	<0.001
***Abdominal obesity***	39.1 (37.8–40.9)	29.0 (26.5–31.5)	47.8 (45.3–50.3)	<0.001
***Prevalence of Full MetS***	35.3	36.6	34.1	0.176

**Note:** Data presented as percentage (confidence interval 95%). Significant at *p*<0.05.

## Discussion

There is some improvement in the prevalence of MetS in the kingdom, particularly in Riyadh region where the study was conducted. Prevalence of MetS in urban areas was previously recorded at 44.1% (1) compared to 37.0% in the present study. The prevalence of dyslipidemia as evidenced by low HDL-cholesterol and high triglyceride levels, however, remained alarmingly high, continuing to be the most common cardiovascular risk factor among Saudi adults. High prevalence of low HDL-cholesterol was also noted among the elderly in Spain [10] and adults in Nigeria [11], but the prevalence is significantly less than that shown in the present study. This calls for further assessment in terms of possible genetic and epigenetic variations within the population. One way to explore this issue is to address genetic determinants of HDL-cholesterol concentrations. Cholesteryl ester transfer protein (CETP), which is detectable at Taq1, was noted to have relatively modest impact on HDL-concentrations among Saudis [12]. The aplolipoprotein B/apolipoprotein A1 ratio on the other hand, was inversely associated to HDL-cholesterol levels and were also associated with components of MetS among Saudis [13]. All in all, little has been done to address the issue of extremely high prevalence of low-HDL cholesterol in the kingdom in terms of identification of causes and early intervention measures. This lack of emphasis has, therefore, translated to the same lack of improvement in terms of decreased prevalence of dyslipidemia in the kingdom, and probably in the extended region as well. Parent-offspring transmission-wise, heritability of MetS components had been previously documented among Omani families with strong tendencies for higher BMI, lower HDL-C and larger body weight while higher triglycerides, diastolic BP, abdominal obesity and insulin resistance were more environmentally driven [14]. Extremely high prevalence of low HDL-C has also been documented among Saudi children, affecting ∼90% of them [15]. Low HDL-C had the highest heritability among Caucasian individuals [16]–[17] and Korean twins [18].

While the prevalence of MetS in males is higher, it is not significantly different from females. Similar findings were found in a recent study by Lin and colleagues, where even if a higher prevalence of MetS among males was observed, the mortality risk was greater among postmenopausal women [19]. Gender differences among the components of MetS are influenced by sex hormones. Indeed, gonadal hormones are directly involved in both glucose and lipid metabolism, with decreased levels of estrogen and/or higher levels of testosterone being associated with insulin resistance and a proatherogenic lipid profile, which makes the male gender by itself a risk factor for cardiovascular disease [20]. Furthermore, this difference may also arise from other factors previously not mentioned which are common among the Saudi male population, such as smoking [21]. Going back to figure 1, males have a higher prevalence of MetS only among the younger age groups, and females eventually catch up once menstruation has ceased, since the cardioprotective effects of the estrogen cycle is lost. Consequently, females have a higher prevalence of obesity than males, and this is true for the rest of the Arabian Gulf States [22].

In summary, the prevalence of the full MetS among urban Saudi adults remains high, but has considerably decreased. Prevalence of low-HDL cholesterol on the other hand remains extremely prevalent and begs for tougher measures in early intervention and further scientific exploration.
